# ﻿*Limnichthyskoreanus*, a new species of creediid fish (Teleostei, Acropomatiformes, Creediidae) from Korea

**DOI:** 10.3897/zookeys.1214.128977

**Published:** 2024-10-01

**Authors:** Yu-Jin Lee, Jin-Koo Kim

**Affiliations:** 1 Department of Marine Biology, Pukyong National University, Busan 48513, Republic of Korea Pukyong National University Busan Republic of Korea

**Keywords:** benthic species, Jeju Island, sand burrower, taxonomy

## Abstract

*Limnichthyskoreanus***sp. nov.** is described on the basis of the holotype and 11 paratypes from subtidal waters of Seogwipo, Jeju Island, Korea. The new species had previously been regarded as the Northern Hemisphere population of the anti-equatorial *L.fasciatus*, but molecular analyses of mitochondrial COI and 16S genes recovers deep genetic divergences of 9.4% and 15.0% between the new species and topotypical specimens of *L.fasciatus*. *Limnichthyskoreanus***sp. nov.** is distinguished from all other species of *Limnichthys* based on the following combination of colouration and morphological characteristics: 38-40 vertebrae; 0–6 dorsal saddles joining mid-lateral stripe; small infraorbital sensory pores; a single median interorbital pore; and well-developed vomerine teeth. Summary characters for comparative congeneric species are provided.

## ﻿Introduction

The family Creediidae consists of 18 species accommodated in eight globally distributed genera, most of which are concentrated in subtropical and tropical coastal waters of the Indo-Pacific Ocean (Nelson et al. 2016; GBIF Secretariat 2023; Fricke et al. 2024). Known colloquially as sand burrowers or sand lancers, creediids are small, slender, sand-dwelling fishes that camouflage and hide in the uppermost layer of sand in wait of passing prey before rapidly darting out and back into the sand in a boomerang fashion (Nelson 1985; Cozzi and Clark 1995). The creediid fishes have the following characteristics: less than 5–6 cm in length; prominent eyes positioned dorsally on the head; fleshy snout; no spines in the dorsal or anal fin rays (Nelson 1985). Studies on *Limnichthys* have compared their morphological phylogenetics and examined skeletal structures, new species, including feeding behavior, and early life history (Nelson 1978, 1979, 1985; Leis 1982; Shimada and Yoshino 1987; Pettigrew and Collin 1995; Yoshino et al. 1999; Pettigrew et al. 2000; Shibukawa 2010; Fricke and Golani 2012). The creediid genus *Limnichthys* is of particular taxonomic interest, with nearly all its species adopting anti-equatorial distribution. The possibility of the existence of cryptic populations has been discussed for a long time (Nelson 1978).

Although creediid species occur far from each other they are highly morphologically similar. Six species of *Limnichthys* are currently recognized as valid: *L.fasciatus* Waite, 1904; L.*marisrubri* Fricke & Golani, 2012; *L.nitidus* Smith, 1958; *L.orientalis* Yoshino, Kon & Odabe, 1999; *L.polyactis* Nelson, 1978; and *L.rendahli* Parrott, 1958. With the exception of *L.nitidus*, which occurs from tropical to temperate water of the Indo-Pacific Ocean, the other species of *Limnichthys* exhibit anti-equatorial distributions (Nelson 1985; GBIF Secretariat 2023). Notably, *L.fasciatus* exhibits a disjunct, anti-equatorial distribution in both hemispheres, but its status as a single, widespread species has not been properly investigated (Nelson, 1978). Morphological and molecular comparisons of specimens identified as *L.fasciatus* from Korea in the Northern Hemisphere indicate that they are different from topotypical examples of *L.fasciatus* from southeastern Australia. Accordingly, we describe *L.koreanus* sp. nov. on the basis of 12 specimens collected during a monitoring survey of subtropical fish species from the subtidal zone of Jeju Island, Korea. The new species is compared with congeneric species, and summary accounts and phylogenetic relationships for species of *Limnichthys* are discussed.

## ﻿Materials and methods

### ﻿Meristics, morphometrics, and specimen deposition

Methods for counting measuring follow Waite (1904), Nelson (1978), and Yoshino et al. (1999). Measurements were recorded to the nearest 0.1 mm using vernier calipers. Morphometric values are summarized in Tables 1, 2, expressed as percentages of the standard length (SL) and head length (HL). Images of specimens were taken using a digital camera (D750; Nikon, Japan). Detailed morphological characteristics were examined with a stereomicroscope (SZX-16; Olympus, Tokyo, Japan).

**Table 1.** Voucher number, institution, collected date, and GenBank number of specimens used in present study.

Species	Voucher Number	*n*	Institution	Collected location (country)	Date	GenBank number	
						COI	16S rRNA
*Limnichthyskoreanus*	MABIK PI00060703 (PKU 63120)	1	National Marine Biodiversity Institute of Korea	Moseulpo, Jeju Island (South Korea)	2022.08.15	OR541978	OR543335
	MABIK PI00060704 (PKU 63121)	1			2022.08.15	OR541979	OR543336
	MABIK PI00060705 (PKU 63122)	1			2022.08.15	OR541980	OR543337
	PKU 21427	1	Pukyong National University		2022.07.13	OR541981	OR543338
	PKU 21528	1			2022.08.15	OR541982	OR543339
	PKU 21529	1			2022.08.15	OR541983	–
	PKU 21530	1			2022.08.15	–	OR543340
	PKU 22426	1			2023.07.17	–	–
	PKU 22427	1			2023.07.17	–	–
	PKU 22428	1			2023.07.17	–	–
	PKU 22626	1		Seongsanpo, Jeju Island (South Korea)	2023.12.16	PP708995	PP708997
	PKU 22627	1			2023.12.16	PP708996	PP708998
*L.fasciatus*	I.44122-031	1	Australian Museum	Central Coast, NSW (Australia)	2007.05.08	OR544519	–
	I.44627-025	1		Nelson Bay, NSW (Australia)	2008.04.08	OR544515	OR543322
	TCWC 17569.03	1	Texas A&M University	Central Coast, NSW (Australia)	2015.02.22	OR544522	OR543331
	CAS 109236*	3	California Academy of Science	Lord Howe Island, NSW (Australia)	1902.12.03 –1903.01.21	–	–
	CAS 38242	3		Lord Howe Island, NSW (Australia)	1973.02.09	–	–
	CAS 120473	7		Sydney, NSW (Australia)	1998.04.15		
	CAS 219288	1		Viti Levu (Fiji)	2002.02.10		
	NSMT-P-125200	1	National Museum of Nature and Science	Japan	–	LC753137	OR543330
	NSMT-P-125201	1		Japan	–	LC753135	OR543329
	NSMT-P-125202	1		Japan	–	OR546102	OR543328
L.cf.nitidus	CAS 228250	5	California Academy of Science	Hawaii Island (USA)	1993.04.15	–	–
	KAUM-I 124455		Kagoshima University	Amami Islands (Japan)	2018.12.16	OR544523	OR543332
	KAUM-I 143956			Amami Islands (Japan)	2020.07.03	OR544524	OR543333
*L.orientalis*	KPM-NI 51923		Kanagawa Prefectural Museum of Natural History	- (Japan)	2010.07.20	LC753149**	–
*Trichonotussetiger*	–		–		–	KU944772**	NC034345**
*T.marleyi*	–				–	JF494737**	–

* Paratypes specimens. ** Sequences were obtained from GenBank.

Osteological details were determined from X-ray images, cleared and stained specimens, and micro-CT data. Micro-CT scans were taken using Phoenix V-Tome-X C450 Volume Graphics® VGSTUDIO Max software and handled using Volume Graphics® myVGL viewer. We performed osteological staining with a paratype specimen (PKU 22428) using a modified version of the method detailed in Song and Parenti (1995). Vertebral counts were presented as a total number of abdominal to caudal vertebrae, which was followed by Nelson (1978). Terminology of skeletal bones followed by Lucena Rosa (1995).

**Figure 1.**
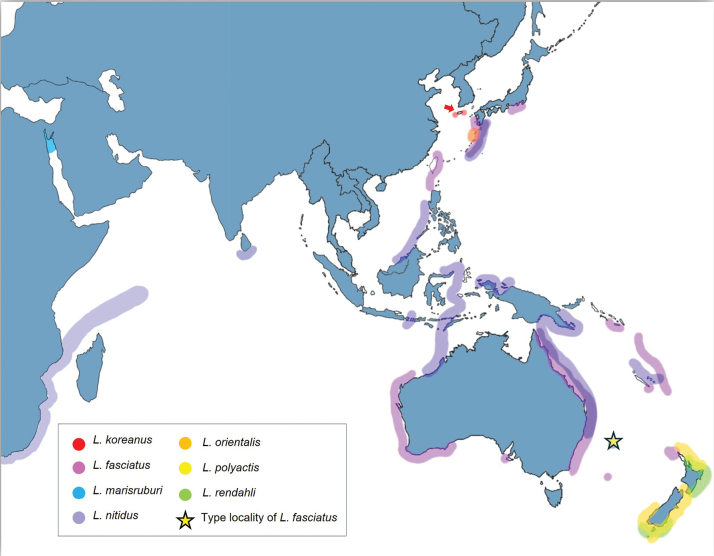
Map showing sampling sites and distribution of *Limnichthys* spp. Each color marks indicate each species: Red marks and arrow showing collected location of *Limnichthyskoreanus* sp. nov.; pink marks showing *L.fasciatus*, which is anti-equatorial species, and yellow star mark showing the type locality of *L.fasciatus*; blue marks showing *Limnichthysmarisrubri* in the Red Sea; purple marks showing *L.nitidus* in Indo-Pacific Ocean; orange marks showing *L.orientalis* in Japan; yellow marks showing *L.polyactis* in New Zealand; green marks showing *L.rendahli* in New Zealand.

Twelve specimens of the new species (35.8–45.3 mm TL, 33.4–40.0 mm SL) were collected from subtidal zones at depths of 1–5 m in Seogwipo, Jeju Island, Korea between July 2022 and December 2023 (Fig. 1). Specimens were vouchered in Pukyong National University (**PKU**) and National Marine Biodiversity Institute of Korea (**MABIK**) with register numbers (PKU 21427; PKU 21528–21530; PKU 22426–22428; PKU 22626–22627; PKU 63120–63122, MABIK PI00060703– MABIK PI00060705). Tissue samples were taken from the right side of the body, preserved in 99% ethanol, and deposited in the **PKU**. We followed the immersion specimen production manual of the **MABIK**, under the National Institute of Marine Biological Resources of the Ministry of Oceans and Fisheries. One specimen was anatomized to investigate the gonadal development. Comparative specimens were loaned and investigated from various museums and institutions, including the Australian Museum, Sydney (**AMS**), the Kagoshima University Museum, Korimoto (**KAUM**), the Kanagawa Prefectural Museum of Natural History, Odawara (**KPM**), Fish Section and Center for Molecular Biodiversity Research, National Museum of Nature and Science, Tokyo (**NSMT**), the California Academy of Sciences, San Francisco (**CAS**), and the Biodiversity Research and Teaching Collections, Department of Wildlife and Fisheries Sciences, Fisheries Sciences, Texas A&M University (**TCWC**). Institutional codes follow Sabaj (2023).

Comparative materials of L.cf.nitidus used in this study were collected from Pacific Ocean (Japan, and Hawaii, USA), which is far from the type locality of the species (Mozambique, South Africa). Given that creediid fishes, in particularly those belonging to the genus *Limnichthys*, exhibit local endemism with the possibility of undescribed cryptic diversity, we refer to these non-topotypical comparative specimens as L.cf.nitidus. X-ray images and micro-CT images of syntypes and non-type material of *L.fasciatus* were examined, as well as skeletal sketch by Nelson (1978). See list of material examined below.

### ﻿DNA extraction, amplification, and sequencing

Genomic DNA was extracted using tissues the AccuPrep Genomic DNA Extraction Kit (Bioneer, Daejeon, Republic of Korea). Mitochondrial cytochrome c oxidase subunit I (COI) and 16S ribosomal RNA (16S) were amplified from extracted gDNA using the polymerase chain reaction. Primer sets for 16S and COI follow by Palumbi (1996) and Ward et al. (2005) respectively. Polymerase chain reaction with a mixture (2 μL 10× buffer, 1.5 μL dNTPs, 2 μL primer set, 0.1 μL Taq polymerase, and 2 μL genomic DNA (gDNA) made up to 20 μL with distilled water) was performed under the following conditions: initial denaturation step at 95 °C for 5 min followed by 35 cycles of denaturation at 95 °C for 30 s, annealing at 52–54 °C for 45 s, and extension at 72 °C for 45 s, with a final extension at 72 °C for 7 min. Amplification was conducted using a thermal cycler (MJ mini PCT-1148; Bio-Rad, Hercules, CA, USA). Sequences were read by BigDye Terminator v. 3.1 cycle sequencing kits (Applied Biosystems, Foster City, CA, USA) with an ABI PRISM 3730XL analyzer (96 capillary type; Applied Biosystems).

### ﻿Molecular analysis

A total number of 5,750 bp in 16S and 7,564 bp in COI sequences were obtained. To analyze the relationships among sequences, alignment was performed using ClustalW (Thompson et al. 1994) in BioEdit 7 (Hall 1999). The final sequence lengths used in the analysis were 401 bp in 16S, and 417 bp in COI per individual. Genetic distances were calculated using Mega 11 (Tamura et al. 2021) with the Kimura 2-parameter (K2P) model (Kimura 1980). Neighbour-joining trees were constructed based on 1,000 bootstrap replications by maximum composite likelihood model using Mega 11 software (Tamura et al. 2004). Sequence data for other species in the genus *Limnichthys* (*Limnichthysorientalis*) and outgroup of two species [Trichonotidae: *Trichonotusmarleyi* (JF494737 in COI); *T.setiger* (NC034345 in 16S; KU944772 in COI)] were obtained from the NCBI database. The accession numbers of all sequences are provided in Table 1.

## ﻿Results

### ﻿Taxonomy

**Acropomatiformes Gill, 1893 (new Korean name: Ban-dit-bul-ge-reu-chi-mok)**

**Creediidae Waite, 1899 (new Korean name: Byeol-ba-ra-gi-gwa)**

**Figure 2.**
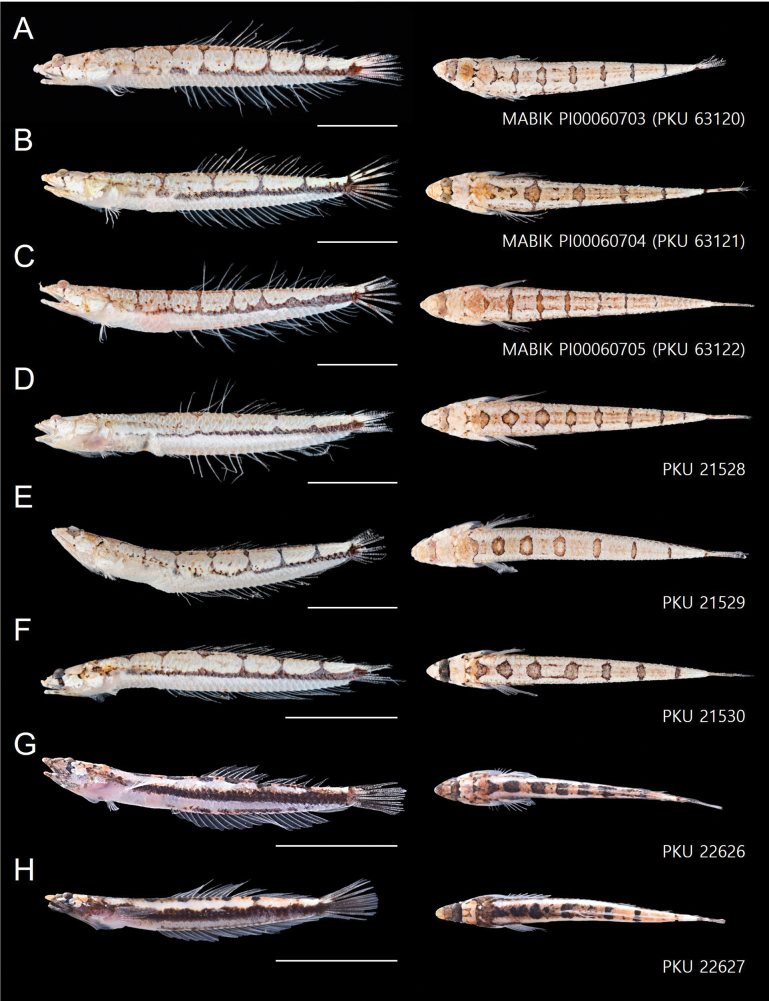
*Limnichthyskoreanus* sp. nov. **A** holotype, MABIK PI00060703 (PKU 63120), 37.3 mm SL, Moseulpo **B** paratype, MABIK PI00060704 (PKU 63121), 38.4 mm SL, Moseulpo **C** paratype, MABIK PI00060705 (PKU 63122), 40.0 mm SL, Moseulpo **D** paratype, PKU 21528, 34.5 mm SL, Moseulpo **E** paratype, PKU 21529, 33.6 mm SL, Moseulpo **F** paratype, PKU 21530, 33.4 mm SL, Moseulpo **G** paratype, PKU 22626, 33.8 mm SL, Seongsanpo **H** paratype, PKU 22627, 35.7 mm SL, Seongsanpo. Scale bars indicate 10 mm. Left images showing lateral views; right images showing dorsal views. Voucher numbers are annotated in the bottom right corner of each image. Scale bars: 10 mm.

**Figure 3.**
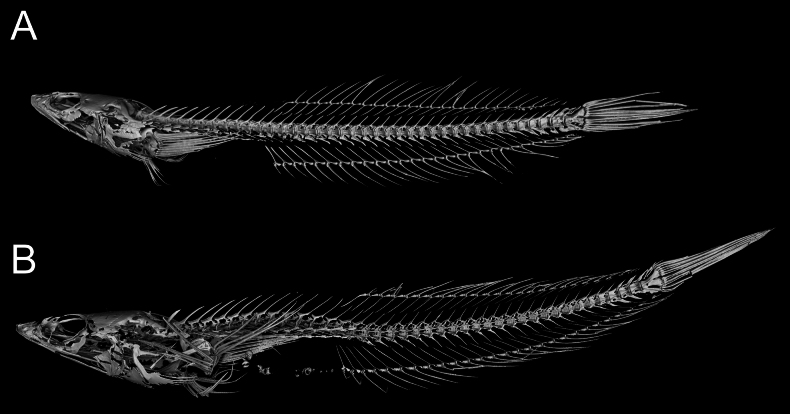
Radiographs of *Limnichthys* species using CT-scan **A***Limnichthyskoreanus* sp. nov., holotype, MABIK PI00060703 (PKU 63120), 37.3 mm SL, South Korea **B***Limnichthysfasciatus*, syntype, AMS I.5858, 43 mm SL, preserved in Australian Museum.

**Figure 4.**
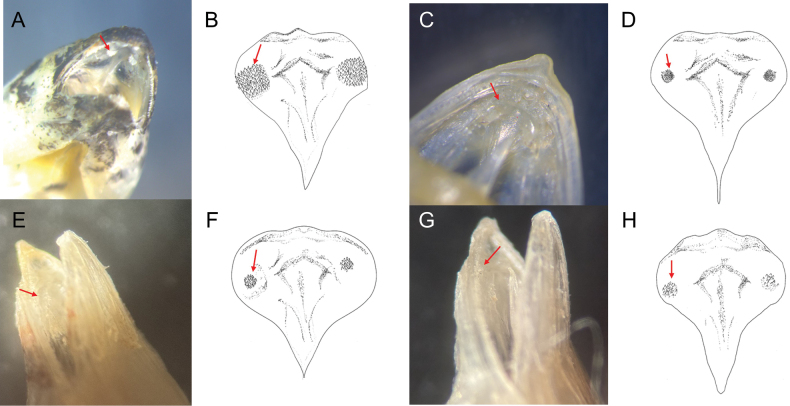
Vomerine teeth of *Limnichthys* spp. The red arrows indicate vomerine teeth. **A, B***Limnichthyskoreanus* sp. nov.; well-developed vomerine teeth; broad and bugling; conical teeth **C, D***L.fasciatus*, weak-developed vomerine teeth; minute conical teeth **E, F**L.cf.nitidus; developed vomerine teeth; narrow and bugling; conical teeth **G, H***L.orientalis*; slightly developed vomerine teeth; conical teeth.

**Figure 5.**
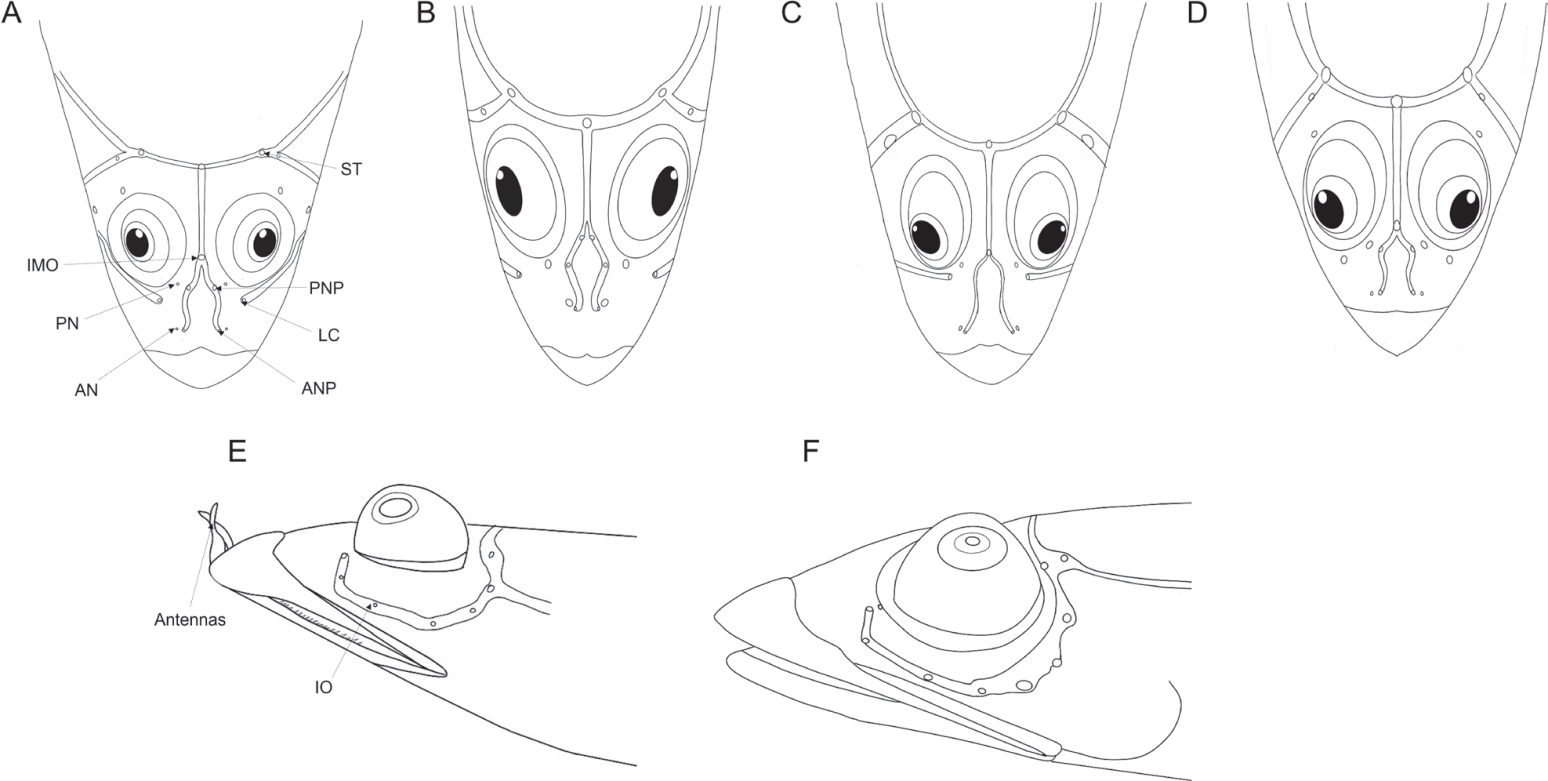
Dorsal and lateral view of head of *Limnichthys* spp. The photos show sensory canals and pores on head **A, E***L.koreanus* sp. nov., sensory canals weak-developed, infraorbital sensory pores small; some species have antennas on snout **B, F***L.fasciatus*; AMS I.44627; Nelson Bay, NSW; 28.7 mm SL; sensory canals well-developed; infraorbital sensory pores large **C**L.cf.nitidus; CAS 228250; Hawaii Island, USA; 20.24 mm SL; sensory canals well-developed **D***L.orientalis*; KPM-NI 51923; Japan; 29.4 mm SL; sensory canals well developed. Abbreviations: AN, anterior nostril; ANP, anterior nasal pore; IMO, interorbital median pore; IO, infraorbital pores; LC, lachrymal pore; PN, posterior nostril; PNP, posterior nasal pore; ST, supratemporal pores.

**Figure 6.**
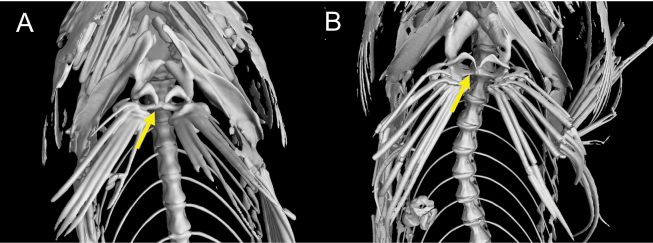
Separation of the anterior process of pelvic girdle **A***Limnichthyskoreanus* sp. nov., holotype, MABIK PI00060703, 39.5 mm SL, South Korea, pelvic girdle well-separated from each other **B***Limnichthysfasciatus*, syntype, AMS I.5858, 43 mm SL, preserved in Australian Museum, pelvic girdle close to each other.

**Figure 7.**
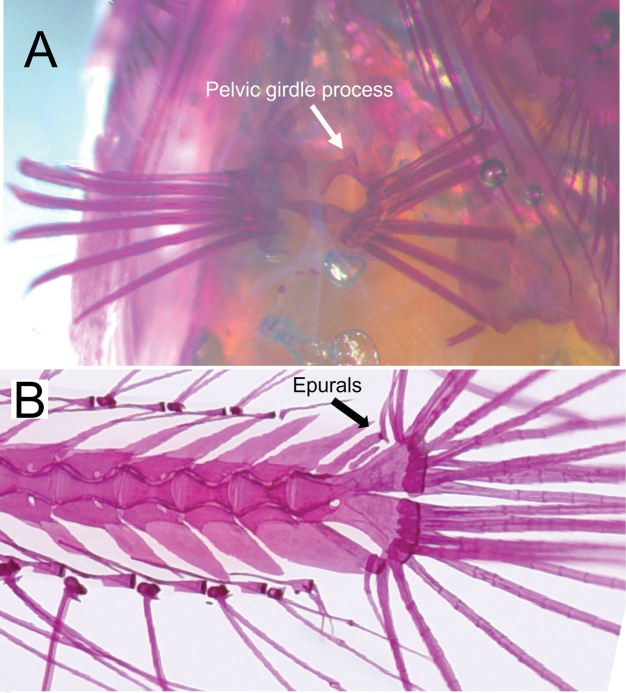
Photos showing detailed morphological traits in a staining specimen of *Limnichthyskoreanus* sp. nov. **A** upper process of pelvic girdle **B** two epurals. Arrows indicate terms of parts.

***Limnichthys* Waite, 1904 (new Korean name: Byeol-ba-ra-gi-sok) (Tables 2, 3, Figs 2–7)**

#### ﻿ Limnichthys koreanus sp. nov.

Taxon classificationAnimaliaPerciformesCreediidae

1B2FE7FC-1AE9-5130-A4B7-05227C8B4801

https://zoobank.org/462BD9BA-4E7A-44AC-96EB-52F8F972771D

Table 1 , 2 , Figs 2 , 3 , 4 , 5 , 6 , 7 New English name: Korean sand burrower; new Korean name: Tti-byeol-ba-ra-gi

##### Material examined.

***Holotype***: South Korea • 45.95 mm TL, 39.5 mm SL; tidal pool on Jeju Island; 33°13'21.1"N, 126°14'30.9"E; 1 m; 15 August 2022; collector Yu-Jin Lee & Jin-Koo Kim; scoop net; MABIK PI00060703 (PKU 63120).

***Paratypes*.** South Korea • 44.5 mm TL, 38.4 mm SL; 15 August 2022; same data as holotype; MABIK PI00060704 (PKU 63121); South Korea • 45.3 mm TL, 40.0 mm SL; 15 August 2022; same data as holotype; MABIK PI00060705 (PKU 63122); South Korea • 1 ♀, 44.5 mm TL, 37.3 mm SL; 14 July 2022; same data as holotype; PKU 21427; South Korea • 38.5 mm TL, 34.5 mm SL; 15 August 2022; same data as holotype; PKU 21528; South Korea • 38.3 mm TL, 33.6 mm SL; 15 August 2022; same data as holotype; PKU 21529; South Korea • 35.8 mm TL, 33.4 mm SL; 15 August 2022; same data as holotype; PKU 21530; South Korea • 42.4 mm TL, 38.5 mm SL; 17 July 2023; same data as holotype; PKU 22426; South Korea • 43.2 mm TL, 37.6 mm SL; 17 July 2023; same data as holotype; PKU 22427; South Korea • 44.1 mm TL, 39.4 mm SL; 17 July 2023; same data as holotype; stain­ing specimen; PKU 22428; South Korea • 37.5 mm TL, 33.8 mm SL; tidal pool on Jeju Island; 33°27'37.0"N, 126°56'02.1"E; 5 m; 15 December 2023; hand net; PKU 22626; South Korea • 38.4 mm TL, 35.7 mm SL; tidal pool on Jeju Island; 33°27'37.0"N, 126°56'02.1"E; 5 m; 15 December 2023; collector Yu-Jin Lee & Jin-Koo Kim; hand net, depth PKU 22627.

##### Diagnosis.

Combined number of dorsal and anal fin rays 52–55; vertebrae 38–40; lateral line scales 42–46; a single median interorbital pore; vomerine teeth well developed; pelvic girdle separated each other; dorsal saddle patterns 5–9; dorsal saddles joining mid-lateral stripe 0–6 (Fig. 3A, Table 2).

**Table 2.** Comparison of counts in *Limnichthys* spp. Parentheses indicate counts of the holotype specimen.

	*L.koreanus* sp. nov.	*L.fasciatus* (Australia & Fiji)	*L.fasciatus* (Japan)	L.cf.nitidus (Japan & Hawaii Is.)	*L.marisrubri* (Red Sea)	*L.orientalis* (Japan)	*L.polyactis* (New Zealand)	*L.rendahli* (New Zealand)
References	In this study	In this study	In this study	In this study Nelson 1978	Fricke and Golani 2012	In this study Yoshino et al. 1999	Nelson 1978	Fricke and Golani 2012
**Number of specimens**	12	19	3	24	22	8	34	–
**Counts**								
Dorsal fin rays	25–27 (25)	24–27	26–27	22–25	22–24	21–23	28–32	29–33
Anal fin rays	26–28 (28)	26–29	27–28	26–28	24–26	24–25	31–34	30–32
Pectoral fin rays	12–13 (12)	11–14	12–13	11–12	13–15	10–11	12–13	13–16
Segment caudal fin rays	8–9 (8)	8	8	8	8	8	8	8
Lateral line scales	42–46 (43)	38–43	43–44	36–38	37–41	41–43	41–46	–
Teeth on vomer	Well-developed	Developed	Developed	Developed	–	Developed	Developed	–
Median interorbital pore	1	2	1	1	–	1	1	–
Total vertebrae	38–40 (40)	40–45	39–41	39–41	–	40–41	43–45	43–45
Number of epural	2	2	2	1	–	1	1	2
Midlateral stripe	Present	Present	Present	Present or absent	Present	Absent	Present	Present
Dorsal saddles (number)	5–9 (8)	7–9	7–9	8–12	11–14	6–11	7–9	6–8
Dorsal saddles joining midlateral stripe (number)	0–6 (4)	5–9	5–6	–	–	–	3–5	3–6

##### Description.

**Figure 8.**
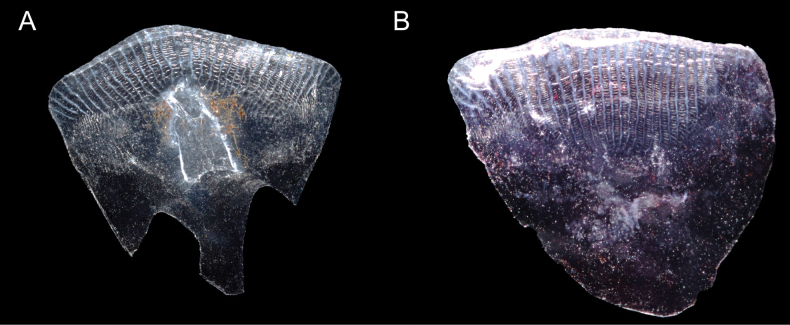
Two types of scales of *Limnichthyskoreanus* sp. nov. **A** pored lateral line scales with trilobed shape **B** a scale on the body. Scales are cycloid.

Counts and measurements of type materials are shown in Table 1; holotype values indicate in parenthesis in table and description. Body elongated; cylindrical and posteriorly compressed. Head to body slope almost flat; head length 24.5–32% in SL (26.1%); head depth 7–10.6% (7.1%); snout length 3.8–5.7% (3.8%) in SL (Table 3). Eyes on dorsal of head, large, and bulging. Snout terminal; upper jaw projects more than lower jaw; upper and lower jaws with a single row of minute conical teeth; a pair of filament-like antennas on anterior upper jaw (or absent); cirri on lower jaw; lips fleshy; vomer with well-developed conical teeth (Fig. 4A); palatine teeth absent; pharyngeal teeth present; tongue slender and pointed. A single of median interorbital sensory pore (Fig. 5A); infraorbital sensory pores very smaller than posterior nostril (Fig. 5E); anterior nostril tubular. Branchiostegal rays 7. Opercular flap covered pectoral fin base. Pectoral fin not reaching anal fin origin; pectoral fin rays 12–13 (12); 6–7^th^ pectoral fin rays longest. Pelvic fin ahead of pectoral fin; 3^rd^ pelvic fin ray longest; pelvic fin not elongated; pelvic fin with I, 5; anterior process of pelvic girdle well separated (Fig. 6A) pelvic girdle with upper projecting process (Fig. 7A). Dorsal fin rays 25–27 (25); Origin of dorsal fin at 3–4^th^ anal fin ray; posterior of dorsal and anal fin reaching precaudal (free from caudal). Anus ahead half of body. Anal fin rays 26–28 (28); anal fin length uniform. Caudal peduncle length very short. All fin rays not branched (only caudal fin branched). Segment of caudal fin rays 8–9 (8). Two epurals (Fig. 6B). Lateral line scales 42–46; lateral line scales trilobed (Fig. 8A); lateral line from opercular to precaudal gradually running down. Body covered with cycloid scales (Fig. 8B); no scales on frontal; scales on cheeks well developed. Gill rakers of first gill arch with small and low multi-spined stubs like patch; gill rakers 2+10.

**Table 3.** Proportional measurements in *Limnichthyskoreanus* sp. nov.

	Holotype (MABIK PI00060703)	Paratypes (*n* = 11)
Standard length (mm)	37.3	33.42–40.0
Morphometric characters		
**In SL (%)**		
Body depth	13.3	10.7–12.8
Head length	26.1	24.5–32.3
Head depth	7.1	7.6–10.6
Snout length	3.8	4.0–5.7
Orbital diameter	3.8	2.6–4.1
Interorbital length	2.1	1.8–2.6
Postorbital length	17.0	15.4–20.2
Upper jaw length	9.7	6.7–11.3
Predorsal length	46.6	44.2–50.5
Prepectoral length	26.8	23.8–30.0
Prepelvic length	23.2	22.3–26.0
Preanal length	41.4	41.9–45.9
Dorsal fin base length	44.0	41.4–49.5
Anal fin base length	52.0	52.9–60.8
**In HL (%)**		
Snout length	16.9	15.2–17.7
Orbital diameter	15.8	9.2–16.7
Interorbital length	4.7	4.4–6.7
Upper jaw length	34.1	24.6–37.7

##### Coloration when fresh.

Body whitish pink. Dorsal and ventral edges pale orange, brown, or white. Dark stripe below eyes. Eyeballs dark brown or black. Opercular pinkish and slightly transparent. Dorsal saddle patterns 5–9, dark brown, dark orange, or black. Distinct horizontal bar on body. Number of dorsal saddle patterns joining with lateral bar 0–6. Pelvic, pectoral, and anal fins transparent. Darkish spots on dorsal fin rays. Caudal fin rays similar in color to body pattern.

##### Coloration when preserved.

Body white. Head white with black or dark brown spots. Dark stripe below eyes. Lateral band black or dark brown. All fins transparent. Spots on dorsal and caudal fin rays. Dorsal or lateral patterns not clearly visible after fixation, depending on preservative solution.

##### Distribution.

The species is presently known only from Jeju Island, Korea.

##### Biology and habitat.

They inhabit relatively thick sand substrates (or maybe more like fine gravels), often hiding almost entirely in the sand in subtidal zone. They tended to dart out to catch prey (e.g. copepods) and then return to their original position. Females have mature eggs in their gonads from June to August. The eggs (522 per individual) are approximately 0.62–0.65 mm in diameter. In contrast, a specimen from December lacked developed gonads.

##### Etymology.

The epithet of the new species, *koreanus*, refers to the type locality (Korea) where the species were collected.

##### Morphological comparisons.

*Limnichthyskoreanus* sp. nov. is clearly distinguished from the other species in the genus *Limnichthys* in having significantly developed vomerine teeth and number of total vertebrae (38–40) (Table 3; Fig. 4). The new species is most similar to *Limnichthysfasciatus*, but can be separated based on the following characters: well-developed vomerine teeth (significant bugling vs weak bugling in *L.fasciatus*); total vertebrae count (38–40 vs 40–45 in *L.fasciatus*); presence of spot pattern on dorsal- and caudal-fin rays (absent in *L.fasciatus*); size of the infraorbital sensory pore below the middle of the eyes (smaller than the posterior nostril [PN] vs similar or larger than the PN in *L.fasciatus*); a single of median interorbital pore (two in *L.fasciatus*) (Fig. 5); separation of the anterior process of pelvic girdle (nearby in *L.fasciatus*) (Fig. 6). Especially, *L.fasciatus* including paratype specimens in the south hemisphere has well-separated interorbital median pores (having two pores in *L.fasciatus* from Lord Howe Island, Fiji, and Sydney). Due to the proximity between the two interorbital median pores, they are often misidentified as one pore. *Limnichthyskoreanus* further differs from L.cf.nitidus, *L.orientalis*, and *L.polyactis* in having: two instead of one epural; dorsal-fin rays (25–27 vs 22–25 in L.cf.nitidus; 21–23 in *L.orientalis*; 28–32 in *L.polyactis*); anal-fin rays (26–28 vs 26–28 in L.cf.nitidus; 24–25 in *L.orientalis*; 31–34 in *L.polyactis*), segement caudal-fin rays (8–9 vs 8); and presence of midlateral body stripe. *L.polyactis* is the endemic species which only distributed in New Zealand, and it is geographically separated from the other species (Fig. 1). *Limnichthysmarisrubri* and *L.rendahli*, which have a single epural, are distinguished following characters: dorsal fin rays (25–27 vs 24–27 in *L.fasciatus* vs 22–24 in *L.marisrubri* vs 29–33 in *L.rendahli*), anal fin rays (26–28 vs 26–29 in *L.fasciatus* vs 24–26 in *L.marisrubri* vs 30–32 in *L.rendahli*).

##### Genetic comparisons.

**Figure 9.**
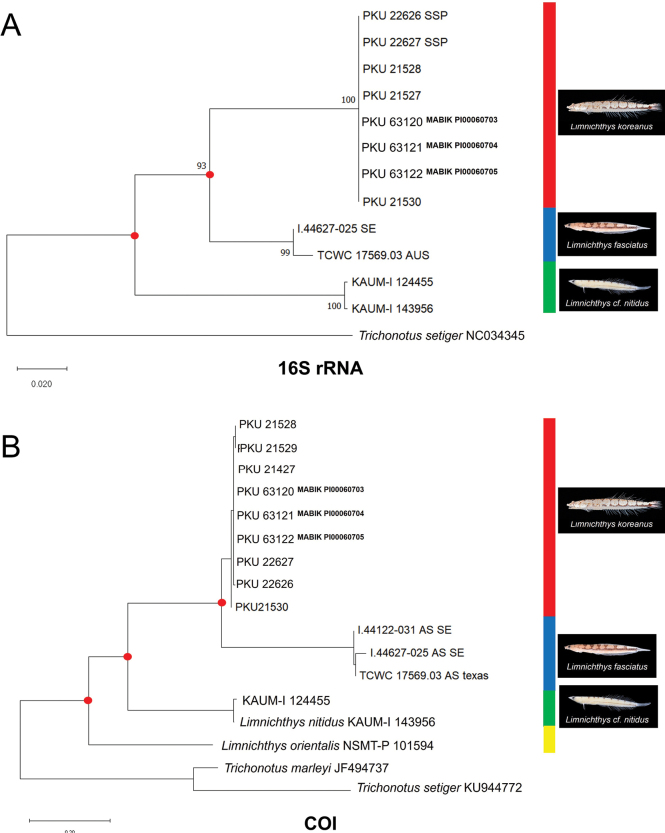
Neighbour-joining tree was constructed based on mtDNA 16S rRNA and COI gene. The tree showing genetic distances among *Limnichthys* species. Bootstrap values based on 1000 replicates were showed 100%, the values are omitted. The red box indicates *Limnichthyskoreanus* sp. nov.; blue box indicates *L.fasciatus* from Southeastern Australia; green boxe indicates L.cf.nitidus from Japan; yellow box indicates *L.orientalis* from Japan; Trichonotidae species was used as outgroup.

COI (510–614 bp) and 16S rRNA (442–508 bp) sequences were obtained from *L.koreanus* sp. nov. After alignment with National Center for Biotechnology Information (NCBI) sequences of other *Limnichthys* species (Fig. 9), we found significant genetic divergences of 9.4% and 15.0% for *L.fasciatus* in the COI and 16S rNA genes from near the type locality (southeastern Australia), respectively. Furthermore, genetic distances of 16.2% and 18.4% were observed for L.cf.nitidus. Only COI gene sequences were obtained for *L.orientalis*, indicating a genetic distance of 20.9%.

## ﻿Discussion

We discovered a new species, *Limnichthyskoreanus* sp. nov., through morphological and molecular analysis of 12 specimens collected from the subtidal zone (at 1–2 m in depth) of Jeju Island, Korea between August 2022 and December 2023. *Limnichthys* species are remarkable because their morphological characteristics are strikingly similar despite high endemism. They commonly have dorsal saddle patterns, and the cirri on the lower jaw are well developed. Initially, this new species was considered a cryptic species of *L.fasciatus* because it appeared to have no morphological differences; it only exhibited a large genetic distance. However, we found significant morphological traits differing from type specimens as follows: the new species has fewer vertebrae; the infraorbital sensory pores below the middle of the eyes smaller than the posterior sensory pores; separation of the anterior process of both pelvic girdles; the highly developed vomerine teeth. Compared with the other species, the new species has spots (rarely absent) on the dorsal fin rays, in contrast to the transparent dorsal fin rays of *L.fasciatus*. The number of dorsal saddle patterns in the new species ranges from 5 to 9, whereas it ranges from 7 to 9 in *L.fasciatus*. Notably, differences in caudal fin ray segments were first discovered in this study. *Limnichthys* species typically have eight caudal fin ray segments and slightly developed vomerine teeth (Nelson 1985; Fricke and Golani 2012). However, our species has 8–9 branched caudal fin rays and well-developed vomerine teeth. Additionally, a previously unreported upper process of pelvic girdle was observed in the new species compared with previous skeletal sketches (Figs 6, 7).

In terms of genetic results, we considered that the individuals used for molecular analysis of *L.fasciatus* were far from the type locality (Lord Howe Island), located 600 km east of Australia. We representatively used three specimens from Nelson Bay and the central coast of New South Wales, the collected location of specimens are about 7–800 km away from each other. We first confirmed that morphological characters of them were perfectly matched with type locality specimens (13 paratypes and 6 specimens from Lord Howe Island), and they have no genetic differences among the three specimens. Therefore, we treated them as truly *L.fasciatus*, which showed deep divergence from our species. Interestingly, we found cryptic diversity in the northwestern Pacific Ocean. *Limnichthysfasciatus* from Japan has similar morphological characteristics to *L.koreanus* sp. nov., but these species have significant genetic divergences. They are well separated from Australian specimens, so they have possibility as a new species. *Limnichthys* species can be distinguished by their distribution, except for *L.nitidus*, and they might have high cryptic diversity and endemism (Fig. 1). *Limnichthysnitidus* is known for having cryptic complex like *L.fasciatus*. According to the original description and subsequent study, the *L.nitidus* complex was treated as subspecies, Indian Ocean species (*L.nitidusnitidus*) and West Pacific Ocean species (*L.nitidusdonalsi*) by ichthyologists (Nelson 1978; Lucena Rosa 1995; Yoshino et al. 1999; Randall 2005). Fricke and Golani (2012) separated each species as *L.nitidus* (Indian Ocean species) and *L.donalsi* (Pacific species), but it is still unclear. For this reason, in this study, we referred to the species as L.cf.nitidus, indicating that it is likely *L.nitidus* but with some uncertainty in the exact identification. Based on ecological traits of *Limnichthys*, such as hiding in the sand and limited mobility, there is a possibility that each region can show high endemism. In the future study, we need molecular comparisons to clarify the genetic populations.

We also noted remarkably prominent color variation among individuals. Specimens of *L.koreanus* sp. nov. collected from western Jeju Island (Moseulpo) were more similar to *L.fasciatus* compared with specimens collected from eastern Jeju Island (Seongsanpo). Nevertheless, mtDNA analysis did not reveal infraspecies sequence variation. We hypothesize that the observed variation in body color of *L.koreanus* sp. nov. is influenced by differences in habitat substrates (bright in the west vs dark in the east), as suggested by Armbruster and Page (1996), who reported that benthic fish with dorsal saddle patterns can vary based on substrate characteristics such as color or particle size. In our study, *Limnichthyskoreanus* sp. nov. was confirmed only in the west (Moseulpo) and east (Seongsanpo) areas of Jeju Island. Future research should investigate its habitat preferences relating to the size of sand (or gravel) for understanding their distribution pattern. All specimens of *L.koreanus* are adults; individuals collected from June to August exhibit maturity for spawning. They are mostly in maturation–mature stages, with an average egg diameter of 0.62–0.65 mm. Creediid fish lay pelagic eggs (Regan 1916; Leis 1982). Specimens were also collected from very shallow coastal waters at depths of 1–5 m. Therefore, spawning may occur near the collection site, highlighting the need for future studies of their spawning and reproductive habitats.

### ﻿Key to species of four species of the genus *Limnichthys* from the West Pacific Ocean

**Table:** 

1	Dorsal fin rays 25 or more; a single epural bone; dorsal saddles joining midlateral stripe absent	**2**
–	Dorsal fin rays 25 or fewer; two epural bones; dorsal saddles joining midlateral stripe present	**3**
2	Pectoral fin rays 11–12; anal fin rays 26–28; lateral line scales 36–38	***L.*** cf. ***nitidus***
–	Pectoral fin rays 10–11; anal fin rays 24–25; lateral line scales 41–43	***L.orientalis***
3	A single interorbital pore; total vertebrae 38–40; dorsal saddles joining midlateral stripe (s) 0–6; vomerine teeth well developed	***L.koreanus* sp. nov.**
–	A pair of interorbital pores; total vertebrae 40–45; dorsal saddles joining midlateral stripes 5–9; vomerine teeth slightly developed	***L.fasciatus***

### ﻿Comparative specimens

*Limnichthysfasciatus*: AMS I.5854; AMS I.5855; AMS I.5856; AMS I.5858, syntypes, 13 specimens, 29–43 mm SL, Lord Howe Island, NSW, Australia; AMS I.44627, 28.7 mm SL, Baronda Headland, south side, NSW, Australia; TCWC 17569, 41.1 mm TL, Forester Beach, Tasman Sea, NSW, Australia.

Limnichthyscf.nitidus: CAS 228250, 5 specimens, 18.44–20.73 mm SL, Hawaii Island, USA; KAUM-I 124455, 21.1 mm TL, 2018.12.16, 31°25'33.0"N, 130°07'11.4"E, south of Kome-jima island, Kataura, Kasasa, Minami-satsuma, Kagoshima, Japan, hand net, collected by Harutaka Hata; KAUM-I 143956, 1 specimen, 21.9 mm TL 2020.06.03, 27°52'03.6"N, 128°58'03.6"E, Daisuke Uyeno, Kagoshima, Japan, Hand net, collected by Ryuichi Nakagawa.

## Supplementary Material

XML Treatment for Limnichthys koreanus

## ﻿Additional information

### Author ORCIDs

Yu-Jin Lee https://orcid.org/0000-0002-9511-0610

Jin-Koo Kim https://orcid.org/0000-0002-8499-406X

### Data availability

The data underpinning the analysis reported in this paper are deposited at GBIF, the Global Biodiversity Information Facility, and are available at https://doi.org/10.15468/a436d4.
